# Effect of Angiotensin II Type 1 Receptor Antagonist, Losartan on Inflammatory Factor in Atherosclerotic Rabbits

**DOI:** 10.5812/cardiovascmed.10781

**Published:** 2013-07-31

**Authors:** Yan-min Xu, Deepak Sharma, Guang-ping Li, Ya-Nan Zhao

**Affiliations:** 1 Department of Cardiology, Tianjin Medical University, Tianjin, Republic of China

**Keywords:** Atherosclerosis, Losartan, C-reactive protein, Interleukin-6, AngiotensinII

## Abstract

**Background:**

Atherosclerosis is a progressive disease characterized by the accumulation of lipids and fibrous elements in the large arteries which now has become the pre-eminent health problem worldwide.

**Objectives:**

To investigate the effect and mechanism of Losartan intervention on atherosclerosis in rabbits fed with high-cholesterol diet.

**Materials and Methods:**

32 New Zealand rabbits were randomly divided into three groups: control group, high-cholesterol group and Losartan group. The level of weights, serum lipid levels and inflammatory factors, such as IL-6 and hs C-reactive protein were detected before the Losartan intervention and two months after the Losartan intervention respectively. The content of AngII was detected on later stage of the experiment. Pathological examination of the iliac arteries was performed to measure the thickness of endothelium and media.

**Results:**

After the atherosclerosis model was established, the level of the serum lipids, hs CRP and IL-6 of rabbits in high-cholesterol group and Losartan group increased significantly in comparison with control group(P < 0.05), but there was no statistical difference between the two groups (P > 0.05). After the Losartan intervention, the levels of serum hs CRP and IL-6 were higher in high-cholesterol group and Losartan group in comparison with control group (P < 0.05), and they were significantly lower in Losartan group than high-cholesterol group (P < 0.05). Serum lipids levels of rabbits in high-cholesterol group and Losartan group also increased significantly in comparison with control group (P < 0.05), but there was no statistical difference between them (P > 0.05). Ratio of endothelium thickness to the media thickness was higher in high-cholesterol group and Losartan group in comparison with control group (P < 0.05), and the ratio in Losartan group was significantly lower than high-cholesterol group (P < 0.05). Content of Angiotensin was higher in high-cholesterol group and Losartan group compared to control group, and there was no statistical difference between them.

**Conclusions:**

The effect of Losartan on atherosclerosis is to prevent the development of atherosclerosis by inhibiting inflammatory process and may not be related to the lipid metabolism.

## 1. Background

Atherosclerosis is a progressive disease characterized by the accumulation of lipids and fibrous elements in the large arteries which now has become the pre-eminent health problem worldwide (1). Over the past few years, we have increased the understanding of the importance of inflammation during all stages of atherosclerosis, from its inception through its progression and its final complication of thrombosis (2, 3). A few studies have showed that the renin-angiotensin system (RAS) participates in the pathogenesis of atherosclerosis (AS) process, angiotensin II(AngII) which is the main active factor in renin-angiotensin system improves the absorption of oxidized low density lipoprotein through a combination with the cell membrane of AngIIreceptor, affects fibrinolysis function of vascular endothelium and inflammation, and thereby accelerates the occurrence and development of AS (4, 5).

## 2. Objectives

In the present study, we explore the effect of Losartan on inflammation factors, such as hs CRP, IL-6 and the level of serum lipids to investigate possible mechanisms of Losartan effects in anti-AS.

## 3. Materials and Methods

### 3.1. Experimental Animals

We declare that there are no human subjects including human material or human data involved. We also clarify that our study (and the protocol used) has been approved by our institutional animal ethics committee. The level of weights, lipids and inflammatory factors, such as IL-6 and hs C-reactive protein and the content of Angiotansin in iliac arteries which were checked in our study have been approved by our institutional animal ethics committee.

 32 healthy purebred New Zealand rabbits weighing from to 2.1 kg, aging from 3 to 4 months were randomly divided into three groups: 1) The control group (n = 11) were on /d of normal diet; 2) high fat group (n = 11 ) were on high /d of cholesterol and saturated fat diet containing 1% cholesterol plus 5% lard plus 10% egg yolk powder and 84% normal diet; 3) Losartan group (n = 10 ) were on the same diet with high fat group.

### 3.2. Experimental Procedure

Rabbits were pre-anaesthetized with xylazine hydrochloride (4 mg/kg IM.). After 20 minutes, anesthesia was induced with a bolus injection of sodium pentobarbital (10 mg/kg IV) and maintained with supplementary injections of 2 ± 4 mg/kg doses as required. Under sterile conditions the right femoral artery was isolated and an arched incision of length in knee region was made. When a femoral arteriotomy was performed, a 0.014 inch guide wire to the artery was sent; thereafter, the balloon was inflated with saline to a pressure of 8-12 atm and inserted with a depth of 12 of artery. The balloon was removed from the artery incision and location of balloon was changed to make intima-injury, the process was repeated for 3 times. The high fat group and Losartan group undergone balloon-injury of right iliac artery intima before feeding. After 4 weeks, all rabbits were weighed and their serum lipid levels and inflammatory markers were measured. Then, the Losartan group were fed 25mg/ kg/d Losartan orally which continued with an 8 week interval. After 12 weeks, the weighing and measurement were repeated. Finally iliac arteries were partially removed and reserved for pathological analysis.

### 3.3. Measurement of Serum Samples

Blood samples were collected at the 4th week and 12th week (8 weeks after losartan intervention). The lipid level was measured using enzymatic method by Selectra-E automatic biochemical analyzer; IL-6 and hs CRP were measured using enzyme-linked immunosorbent assay (ELISA) kits. AngII was measured using radioimmunoassay kits.

### 3.4. Determination of Intima to Media Thickness Ratio

All vessel sections were stained with haematoxylin and eosin. Measurements were made of cross-sectional thickness of the lumen and enclosed by the internal and external elastic laminar. The intimal cross-sectional thickness of femoral artery segments was determined by subtracting the thickness of the lumen from the thickness enclosed by the internal elastic lamina. The medial thickness was determined by subtracting the thickness enclosed by the internal elastic lamina from the thickness enclosed by the external elastic lamina. The Image-Pro Plus 6.0 image analysis system was used to measure intima and media thickness and calculate the ratio.

### 3.5. Statistical Analysis

All data processing statistics are used SPSS 13.0. Continuous variables are expressed as mean ± standard deviation. Statistical analysis was performed by one-way analysis of variance (ANOVA) among groups. For comparison of data before and after dietary and drug intervention, a paired t test was used. We considered p < 0.05 to be statistically significant.

## 4. Results

### 4.1. Effects of Losartan on Serum Lipid

In the fourth and twelfth week, the serum lipid of rabbits in high fat group and Losartan group increased significantly compared with control group (P < 0.05). But there was no statistical difference between the two groups. Serum lipid levels at the twelfth week were higher than the fourth week in high fat group and Losartan group (P < 0.05) (Table 1).

**Table 1.** The TG, TC and LDL-C Levels of Serum in Different Groups and Different Periods (mmol/L, means = SD)

	Control Group (n=11)	High Lipid Group (n=11)	Losartan Group (n=10)
**4 ± TG**	0.82± 0.20	12.89± 8.35^a^	12.88± 9.58^a^
**TC**	1.77 ± 0.62	51.98 ± 15.84^a^	45.23 ± 15.80^a,b^
**LDL-C**	1.02 ± 0.60	22.56 ± 6.92^a^	19.61 ± 5.95^a,b^
**12 ± TG**	0.85 ± 0.27	27.72 ± 18.44^a^	24.88 ± 15.07^a,[Table-fn fn2980]b^
**TC**	1.70 ± 0.64	69.81 ± 18.24^a^	67.84 ± 13.72^a,b^
**LDL-C**	1.04 ± 0.60	29.59 ± 7.87^a^	29.84 ± 7.56^a,b^

^a^Compared with Control Group, P < 0.05

^b^Compared With High Fat Group, P > 0.05

### 4.2. Effects of Losartan on IL-6 and hs CRP

In the fourth week, IL-6 and hs CRP levels of rabbits in high fat group and Losartan group increased significantly in comparison with control group (P < 0.05). But there is no statistical difference between the two groups. In the twelfth week, IL-6 and hs CRP level of rabbits in high fat group and Losartan group also increased significantly in comparison with control group (P < 0.05). But IL-6 and hs CRP level in Losartan group was lower than high fat group (P < 0.05). IL-6 and hs CRP levels at the twelfth week were lower than the fourth week in Losartan group (P < 0.05). But IL-6 and hs CRP levels at the twelfth week had no significant difference compared with the fourth week in high fat group and control group (P > 0.05) (Table 2).

**Table 2.** The Level of hs CRP and IL-6 in Different Groups of Different Periods (mean ± SD)

	Control Group (n=11)	High Lipid Group (n=11)	Losartan Group (n=10)
**CRP (μg/ml) 4 w**	2.86 ± 0.38	4.30 ± 0.32^a^	4.53 ± 0.36^a,b^
**12 w**	2.87 ± 0.34	4.60 ± 0.33^a^	3.34 ± 0.38^a,c^
**IL-6 (pg/ml) 4 w**	19.10 ± 19.16	98.00 ± 19.56^a^	101.78 ± 16.56^a,b^
**12 w**	27.00 ± 18.14	97.40 ± 18.41^a^	58.56 ± 13.06^a,c^

^a^Compared with Control Group; P < 0.05

^b^Compared with High Fat Group, P > 0.05

^c^Compared with High Fat Group, P < 0.05

### 4.3. Effects of Losartan on AngII Level

AngII level was higher in high fat group and Losartan group in comparison with control group (P < 0.05). However, there were no statistical differences between the two groups, discussed above (P > 0.05) (Table 3).

**Table 3.** The Level of AngII in Vascular Tissue (pg/mg mean ± SD)

	Control Group (n=11)	High Fat Group (n=11)	Losartan Group (n=10)
**Ang II**	73.397 ± 9.511	134.367 ± 60.244^a^	133.979 ± 62.440^a,b^

^a^Compared with Control Group, P < 0.05

^b^Compared with High Fat Group, P > 0.05

### 4.4. Effect of Losartan on the Ratio of Intima Thickness

**Figure 1.**
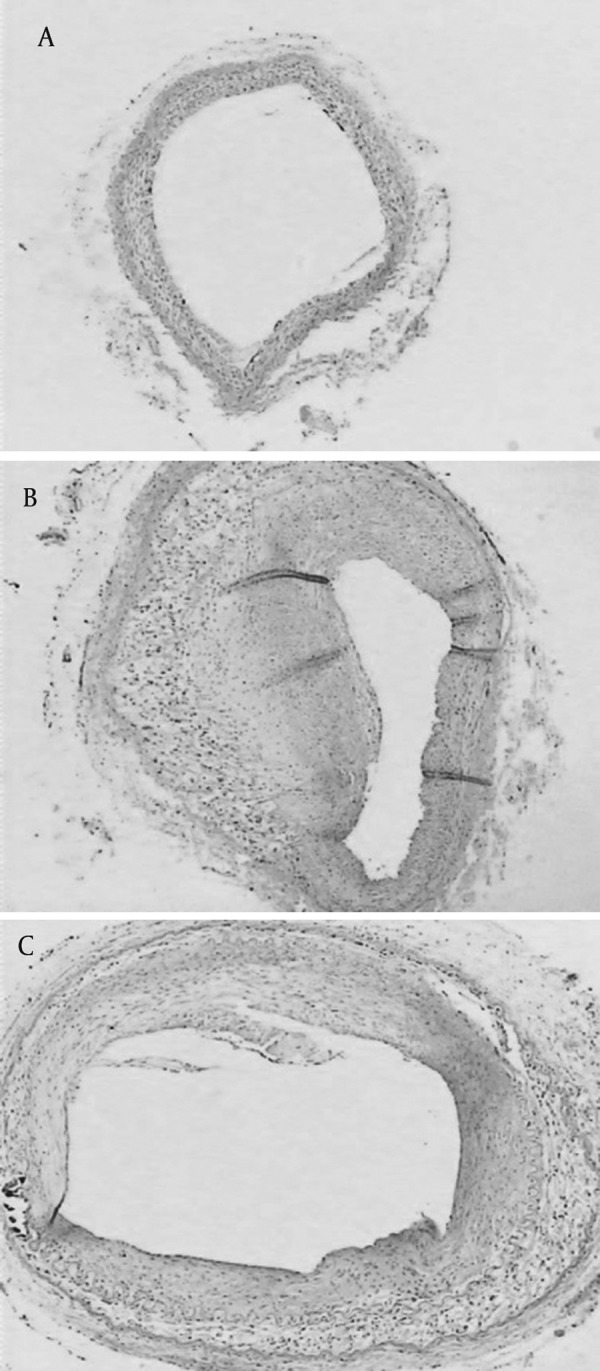
Ratio of Intima Thickness to the Media Thickness

Processing of image analysis system showed that the ratio of intima thickness to the media thickness were higher in high fat group and Losartan group, compared to control group (P < 0.05), and the ratio in Losartan group was lower than high fat group (P < 0.05) (Table 4), (Figure 1).In left is the control group, in middle is high fat group and in right is Losartan group. The ratio in Losartan group was lower than high fat group.

**Table 4.** The Ratio of Intima to the Media of Three Groups (mean ± SD)

	Control Group	High Fat Group	Losartan Group
**Intima/media**	0.208 ± 0.082	1.864 ± 0.556^a^	1.098 ± 0.493^b^

^a^Compared with Control Group, P < 0.05

^b^Compared With High Fat Group, P < 0.05

## 5. Discussion

Renin-angiotensin-aldosterone (RAA) system is the complex neuroendocrine system and regulates vascular tone and salt metabolic balance. Studies have shown that RAA system exists in local vascular and in many pathological situations its activity increases (6-8). AngII on vascular wall cells and extracellular matrix, changes the expression of the inflammatory cytokines, chemokines and adhesion molecules, leading to endothelial dysfunction, promotes lipid deposition and inflammation, eventually leading to the formation of atherosclerosis (9, 10). 

This study shows that high fat group has obvious plaque on arterial wall, lumen narrowing and the elastic membrane stretching and fracturing. Outer membrane did not change. The ratio of intima to media is nine times than the ratio of the control group. The degree of atherosclerosis in Losartan group was alleviated compared with high fat group. The ratio of intima to media was reduced by about 1 time compared with the high fat group. In the current study for the first time, we demonstrate that treatment with the Losartan may play a role against atherosclerosis. 

At the end of the treatment period, we observed a significant increase of serum lipid and AngII in high fat group and Losartan group than the one in control group, but there was no difference between the two groups. We also observed a significant decrease in the level of blood hs CRP and IL-6, a pro-inflammatory cytokines in Losartan group. Therefore, the results suggest that hyperlipidemia activates RAAS and increases AngII to promote the formation of atherosclerosis and also suggest that the anti-atherosclerosis effects of Losartan does not include reducing AngII and lipid-lowing effect , but an there exists an anti-inflammatory effect of Losartan the atheroclerosis model rabbits. These results are in disagreement with previous studies (11, 12). Previous studies indicate that the blood pressure elevation and endothelial dysfunction are associated with atherosclerosis on RAS signaling pathway. Data showed that, NF-κB can regulate various inflammatory reactions and gene transcription, producing a variety of pro-inflammatory factors (13, 14).

Losartan may be acting on inhibiting protein of nuclear factor κB and lead MCP-1, VCAM-1, E-selection in adhesion process to reduce, inhibiting the inflammatory response in occurrence of atherosclerosis (15, 16). Hence, further study is required to be done. As our study, we assume, the inhibiting inflammatory process is the primary function of Losartan on atherosclerosis to prevent the development of atherosclerosis .In short, the formation of atherosclerosis is the result of multiple factors. AT1 receptor antagonist (Losartan) not only reduces blood pressure by reverse ventricular remodeling but also inhibits the occurrence and development of atherosclerosis. The effect of Losartan on atherosclerosis is to prevent the development of atherosclerosis by inhibiting inflammatory process and may not be related to the lipid metabolism.
